# Cytogenetic Analysis of the South American Fruit Fly *Anastrepha fraterculus* (Diptera:Tephritidae) Species Complex: Construction of Detailed Photographic Polytene Chromosome Maps of the Argentinian *Af*. *sp*.*1* Member

**DOI:** 10.1371/journal.pone.0157192

**Published:** 2016-06-30

**Authors:** Angeliki Gariou-Papalexiou, María Cecilia Giardini, Antonios A. Augustinos, Elena Drosopoulou, Silvia B. Lanzavecchia, Jorge L. Cladera, Carlos Caceres, Kostas Bourtzis, Penelope Mavragani-Tsipidou, Antigone Zacharopoulou

**Affiliations:** 1 Biology Department, University of Patras, Patras, Greece; 2 Instituto de Genética EA Favret, Instituto Nacional Tecnología Agropecuaria, Hurlingham, Provincia de Buenos Aires, Argentina; 3 Insect Pest Control Laboratory, Joint FAO/IAEA Division of Nuclear Techniques in Food and Agriculture, Seibersdorf, Vienna, Austria; 4 Department of Genetics, Development and Molecular Biology, School of Biology, Faculty of Sciences, Aristotle University of Thessaloniki, Thessaloniki, Greece; Virginia Tech, UNITED STATES

## Abstract

Genetic and cytogenetic studies constitute a significant basis for understanding the biology of insect pests and the design and the construction of genetic tools for biological control strategies. *Anastrepha fraterculus* is an important pest of the Tephritidae family. It is distributed from southern Texas through eastern Mexico, Central America and South America causing significant crop damage and economic losses. Currently it is considered as a species complex; until now seven members have been described based on multidisciplinary approaches. Here we report the cytogenetic analysis of an Argentinian population characterized as *Af*. *sp*.*1* member of the *Anastrepha fraterculus* species complex. The mitotic karyotype and the first detailed photographic maps of the salivary gland polytene chromosomes are presented. The mitotic metaphase complement consists of six (6) pairs of chromosomes, including one pair of heteromorphic sex chromosomes, with the male being the heterogametic sex. The analysis of the salivary gland polytene complement shows a total number of five long chromosomes that correspond to the five autosomes of the mitotic karyotype and a heterochromatic network corresponding to the sex chromosomes. Comparison of the polytene chromosome maps between this species and *Anastrepha ludens* shows significant similarity. The polytene maps presented here are suitable for cytogenetic studies that could shed light on the species limits within this species complex and support the development of genetic tools for sterile insect technique (SIT) applications.

## Introduction

Cytogenetic analysis of Diptera species has been greatly facilitated by the existence of polytene chromosomes. Since the first publication of the chromosome maps of *Drosophila* [1], polytene chromosomes have proven to be an excellent genetic tool for studying chromosome structure and function, gene activity, phylogenetic relationships and have served as diagnostic tools for distinguishing members of species complexes [2–8]. Moreover, they provide means for detailed cytogenetic maps through the precise mapping by *in situ* hybridization [9].

For insect pest species belonging to the family of Tephritidae, advances in the field of cytogenetics contributed in understanding variation, evolution and incipient speciation phenomena as well as developing and improving pest control methods. The polytene chromosome maps of the Mediterranean fruit fly *Ceratitis capitata* [10,11] helped to improve the Sterile Insect Technique (SIT) by supporting the development of Genetic Sexing Strains (GSS), reviewed in [12–14]. Therefore it is considered as a tephritid model species for the development of GSS through classical genetic approaches and SIT aplications. Similarly, cytogenetic analysis of mitotic and polytene chromosome maps have helped the analysis of GSSs in other tephritid species, such as *Bactrocera dorsalis*, *B*. *cucurbitae* [15] and *Anastrepha ludens* [16].

The Tephritidae family includes five genera (*Anastrepha*, *Bactrocera*, *Ceratitis*, *Dacus* and *Rhagoletis*) of frugivorous species that oviposit eggs in fruits and the developing larvae feed on the mesocarp. About 100 of the tephritid species are of major economic importance. The *Anastrepha* genus is endemic to tropical and subtropical regions of American Continent. Currently, approximately 200 species have been identified, distributed in 17 intrageneric groups. The *A*. *fraterculus* group includes 29 species and most of them occur in Brazil [17–19].

The *A*. *fraterculus* species complex attacks more than 80 plant species, including major fruit crops [20]. It has been reported from southern Texas to Mexico, Central and South America [17,21,22]. Early studies showed differences among populations regarding morphology/morphometry [21], host preference [23,24], isozyme profiles [25] and mitotic karyotypes [26,27]. These early studies led to the assumption that the nominal *A*. *fraterculus* is a species complex. Recent studies have clearly shown that the resolution of species complexes must be based on a multidisciplinary approach, utilizing different and independent lines of evidence [28–30]. In this respect, a variety of tools have been used to shed light to the species limits among the entities of the *A*. *fraterculus* complex. These include studies on morphometrics [31–35], pre- and post-zygotic isolation [36–43] metaphase karyotypes [34,44,45], egg morphology and embryonic development [46–49], DNA markers [50–52] and pheromone profiles [53–56]. Some of the more recent studies have tried to incorporate multidisciplinary approaches for the same samples [32,57,58]. All these studies support the earlier observations about this species complex and provide insight regarding the relationships and limits among its taxa. Until now seven (7) distinct entities (*Af*. *sp*.*1-7*) have been identified and their geographic distribution has been described [33,37,41].

Regarding cytogenetics, different studies attribute specific mitotic karyotypes to the different entities of this complex, based on differences restricted to sex chromosomes [32,45,57,59]. In respect to the polytene chromosomes, previous efforts have presented photographs of polytene elements [60] which, however, have not provided a complete and workable polytene chromosome map. Polytene chromosomes were also used, combined with other approaches, for the analysis of two *A*. *fraterculus* populations as well as their hybrids [57]. This study revealed differences in mitotic karyotype and a high level of asynapses of polytene chromosomes in their hybrids. The cytogenetic work previously performed for this complex has been recently reviewed [61,62].

Here we present the metaphase karyotype and the first detailed photographic polytene chromosome maps from salivary glands of the Argentinian *A*. *fraterculus Af*. *sp*.*1* member of the complex. These maps can be used as reference material for future phylogenetic studies on the *A*. *fraterculus* complex and other *Anastrepha* species. They can also support the construction and characterization of GSS for SIT purposes and facilitate genome mapping of the species, if coupled with *in situ* hybridization experiments.

## Material and Methods

### *Anastrepha fraterculus* strain

A laboratory colony of *Af*. *sp*.*1* maintained at the Joint FAO/IAEA Insect Pest Control Laboratory (IPCL) was used in this study. This strain was derived from pupae sent from the Estacion Experimental AgroIndustrial Obispo Colombres, Tucuman, Argentina. The history of the strain is described in [42]. The colony is kept in standard adult (1 yeast: 3 sugar) and larval carrot diet (7% brewer’s yeast, 0.25% sodium benzoate, 0.2% methylparaben, 0/8% (v/w) HCl, 15% carrot powder, all dissolved in water).

### Mitotic chromosome preparations

Spread chromosome preparations were made from brain ganglia of third–instar larvae using the method reported for *C*. *capitata* [11,63]. Brain tissue was dissected in Ringer’s solution and transferred to hypotonic solution (1% sodium citrate) on a depression slide for 10–15 min and then fixed for 3 min in freshly prepared fixative (3:1 methanol–acetic acid). During this step the fixative was changed at least two times to ensure the complete removal of the water. By the end of the fixation, the fixative was removed and a small drop of 60% acetic acid was added. Working quickly, the tissue was dispersed by drawing up into a micropipette for several times. The cell suspension was finally laid on a clean slide on a warm hotplate (40°–45°C) for drying. Chromosomes were stained with 5% Giemsa in 10mM phosphate buffer, pH 6.8. More than 15 slides prepared from about 30 larvae were analyzed in phase contrast microscope (LEIKA DMR) using 100X objective and the well spread metaphases were photographed using a digital camera (ProgResCF^cool^ JENOPTIC/JENA).

**C-banding**: C-banding was performed as described in [63,64].

### Polytene chromosome preparations

Polytene chromosome preparations were made from well fed third-instar larvae or 1–2 days old pupae [11,63,65]. Larvae were dissected quickly in 45% acetic acid and salivary glands were carefully transferred to 3N HCl on a depression slide for 1 min. Glands were fixed in glacial acetic acid: water: lactic acid (3:2:1) for about 5 min before staining in lacto- acetic- orcein for 5–7 min. Early pupae were dissected in Ringer’s solution and the glands were transferred to 45% acetic acid for 2–3 min and then fixed in 1N HCl for 2 min. The material was passed through lacto acetic acid (80% lactic acid:60% acetic acid, 1:1) and stain in lacto acetic orcein for 10–20 min. Excess stain was removed by washing the glands in lacto-acetic acid before squashing. Chromosome slides were analyzed at 60X and 100X objectives in a phase contrast microscope (LEIKA DMR). Well spread nuclei or isolated chromosomes were photographed using a digital camera (see above). A significant number of chromosome slides were prepared from 500 larvae or pupae and the best of them with well spread nuclei (at least 200 slides) were used for analysis.

### Construction of photographic polytene maps

Photographs showing well spread nuclei and/or isolated chromosomes of sufficient banding pattern quality, were selected and used. The first step was to select chromosomal regions belonging to each chromosome that: a) provided a clear banding pattern and, b) could unambiguously demonstrate the continuity of each polytene element. Afterwards, selected chromosomal regions were assembled using the Adobe Photoshop CS6 Extended Software, to construct the composite photographic map for each chromosomal element.

## Results

### Mitotic chromosomes

**Fig 1:**
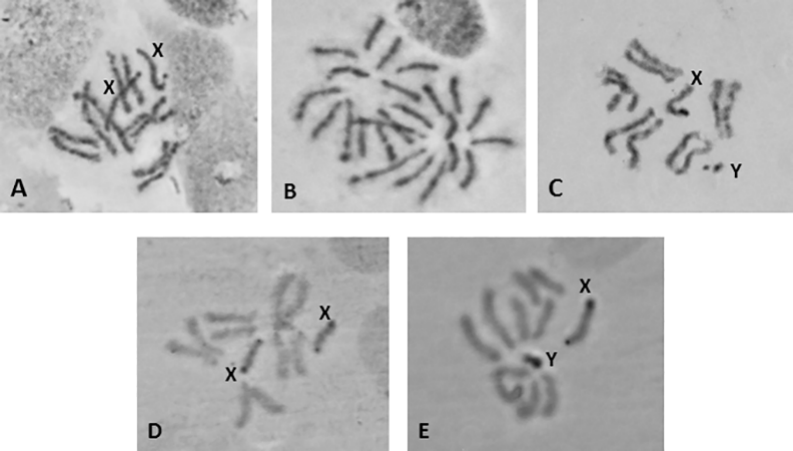
Mitotic karyotype of *A*. *fraterculus*. (A, B, D) female; (C, E) male. (A, B, C) Giemsa staining; (D, E) C-banding. The sex chromosomes, X and Y, are shown. The acrocentric nature of the chromosomes is evident in (B).

The analyzed Argentinian strain of *A*. *fraterculus* has six pairs of chromosomes including five pairs of autosomes and one pair of sex chromosomes, with the male being the heterogametic sex (XY). Fig 1 shows chromosome spreads derived from both male (1C, E) and female (1A, B, D) larvae. All the chromosomal elements are acrocentric with the exception of the Y chromosome which is probably submetacentric [45]. Two of the autosomes are longer and are easily distinguished from the rest, which are more or less of similar size. Both sex chromosomes are highly heterochromatic as shown following Giemsa (Fig 1A, 1B and 1C) and C-banding staining (Fig 1D and 1E), in accordance with previous studies based also on Giemsa staining and C banding [57,66]. From (Fig 1A, 1C, 1D and 1E) it is clear that autosomes present two chromatids, while sex chromosomes do not show two chromatids. This is probably related to the late replication of sex chromosomes, which in turn is supportive of their heterochromatic nature (Bedo 1987). The labelling system is based on that proposed by Radu and colleagues [67] for *C*. *capitata*, the first analyzed species of the Tephritidae family. The sex chromosomes are labeled as the first pair of the mitotic karyotype and the autosomes from 2–6 in order of descending size. This karyotype is in full agreement with that of the *A*. *sp*. *1* member of the complex [45,59].

### Polytene chromosomes

**Fig 2:**
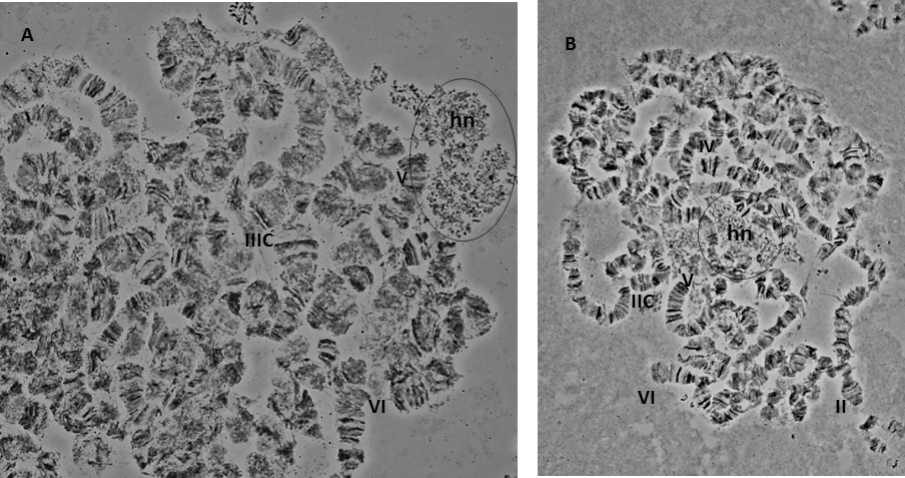
Heterochromatic network (hn) representing the under-replicated sex chromosomes. The heterochromatic network (hn) is indicated. Selected telomeres and centromeres are marked in the two nuclei.

The polytene chromosomes of *A*. *fraterculus* are not an easy material to work with, due to a variety of reasons: a) polytene elements are long due to their acrocentric nature, b) the lack of a typical chromocenter complicates the location of the centromere for each element, c) the frequent chromosome fragmentation makes the analysis difficult and d) most of the chromosomal regions have a poor banding pattern and this combined with their tight coiling and twisting further compromises the identification of each element. However, these difficulties were overcome using and combining a large number of selected photographs to achieve the results presented here. The analysis showed that *A*. *fraterculus* polytene complement consists of a total of five long elements that correspond to the five autosomes, in agreement to the acrocentric nature of the mitotic complement. Sex chromosomes do not form polytene elements because of their heterochromatic nature. Their presence in polytene nuclei is evident by a heterochromatic network (Fig 2A and 2B).

**Fig 3:**
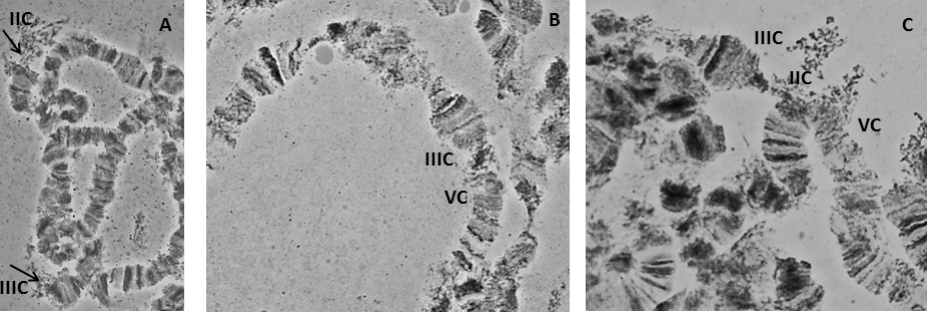
Centromeric regions of *A*. *fraterculus* polytene chromosomes. (A) Centromeres of chromosomes II and III, (B) a partial chromocenter involving chromosomes III and V, (C) a partial chromocenter involving three chromosomes, II, III and V. Arrows indicate the heterochromatic threads in (A). C indicates the centromere.

**Fig 4:**
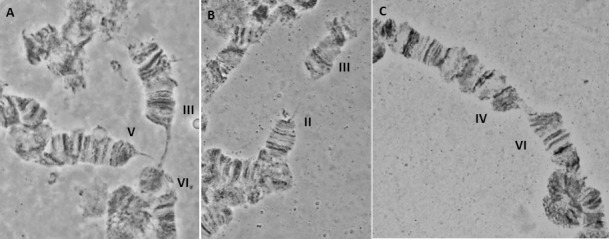
Ectopic pairing between telomeres of *A*. *fraterculus* polytene chromosomes. (A) a three-way pairing between telomeres of chromosomes III, V and VI, (B) II and III chromosomes, (C) IV and VI chromosomes.

In species lacking a chromocenter, several criteria have been used for localizing the centromeres and subsequently the free end (telomere) [10,11,68,69]. Centromeric positions usually appeared as weak points or constrictions, as well as regions (bands) with heterochromatic nature. In the case of the *A*. *fraterculus*, we observed characteristic structures that most likely represent centromeric regions, such as heterochromatic threads which are connected to some chromosome ends (Fig 3A). Moreover, there are cases where more than one chromosomes are connected to these heterochromatic structures giving the impression of a partial chromocenter (Fig 3B and 3C). An additional characteristic of the polytene chromosomes of *A*. *fraterculus* is the ectopic pairing between chromosomes ends that, interestingly, are never connected to the previous heterochromatic threads suggesting that they represent the telomeres of the chromosomes (Fig 4A–4C). Such phenomena were also observed in the analysis of *A*. *ludens* [68].

**Fig 5:**
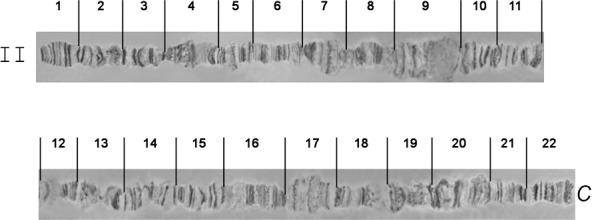
Photographic map of the *A*. *fraterculus (A*.*sp*.*1)* salivary gland polytene chromosome II (sections 1–22). C indicates the centromere.

**Fig 6:**
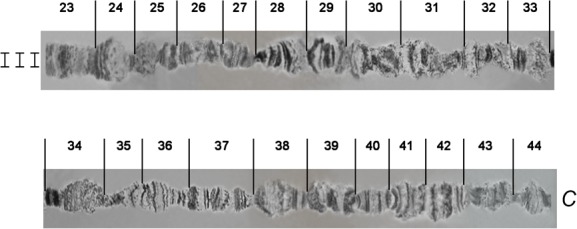
Photographic map of the *A*. *fraterculus (A*.*sp*.*1)* salivary gland polytene chromosome III (sections 23–44). C indicates the centromere.

**Fig 7:**
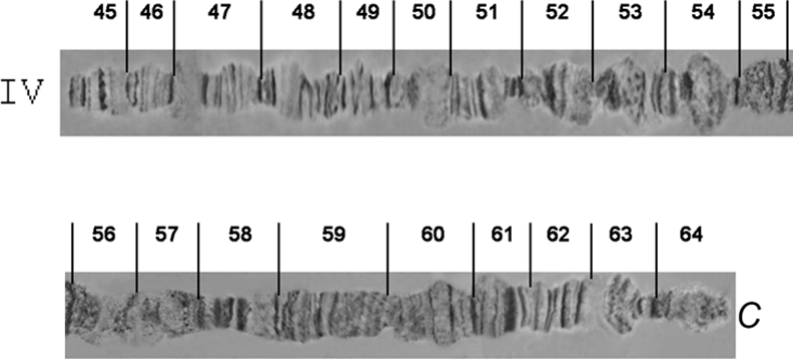
Photographic map of the *A*. *fraterculus (A*.*sp*.*1)* salivary gland polytene chromosome IV (sections 45–64). C indicates the centromere.

**Fig 8:**
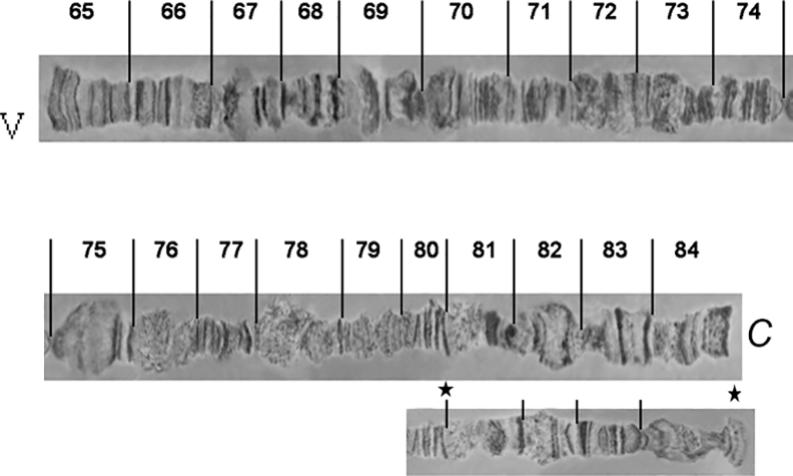
Photographic map of the *A*. *fraterculus (A*.*sp*.*1)* salivary gland polytene chromosome V (sections 65–84). Asterisks indicate an alternative appearance of chromosomal region 81–84, due to differences in puffing pattern. C indicates the centromere.

**Fig 9:**
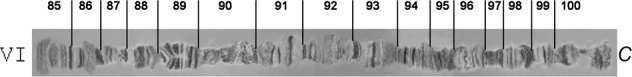
Photographic map of the *A*. *fraterculus (A*.*sp*.*1)* salivary gland polytene chromosome VI (sections 85–100). C indicates the centromere.

The *A*. *fraterculus* polytene chromosome reference maps are shown in Figs 5–9. Chromosomes are labelled from II to VI according to their size, following the numbering system used for the first analyzed *Anastrepha* species, *A*. *ludens*. It is necessary to emphasize that this labeling does not imply any correlation to the mitotic karyotype. Sex chromosomes, which are not polytenized, are not represented in the polytene complement. The whole polytene complement was subdivided into 100 sections taking into account the most prominent or distinctive bands as section boundaries. The most prominent diagnostic landmarks for each element are given below.

### Chromosome II, sections 1–22 (Fig 5)

**Fig 10:**
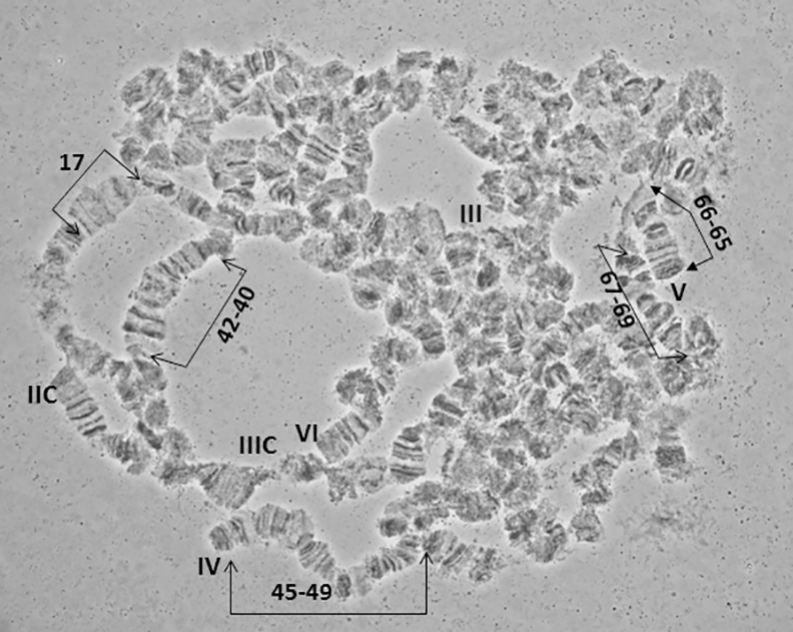
A polytene nucleus of *A*. *fraterculus*. Characteristic landmarks of different polytene chromosome arms are shown. Sections 17, 40–42, 45–49 and 65–69 are indicated. Four of the five telomeres (III, IV, V, VI) and two of the five centromeres (IIC, IIIC) are also noted.

**Fig 11:**
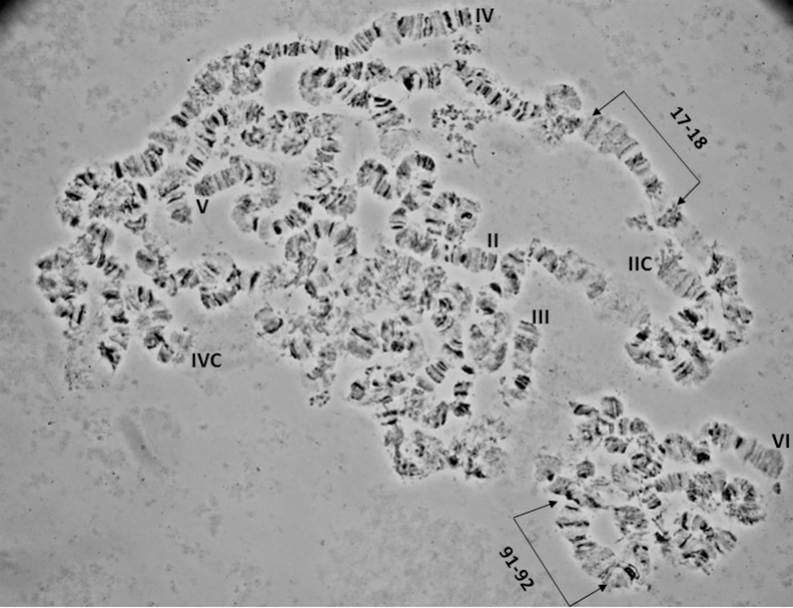
A polytene nucleus of *A*. *fraterculus*. Characteristic landmarks of different polytene chromosome arms are shown. Sections 17–18 and 91–92 are indicated. The five telomeres (II, III, IV, V, VI) and two of the five centromeres (IIC, IVC) are also noted.

Chromosome II is slightly longer than chromosome III and is easily identified because of the two characteristic ends, the telomere in section 1 and the proximal to the centromere region in section 22. The teleomere usually participates in ectopic pairing with other telomeres (Fig 4B). The centromere very often carries heterochromatic threads or participates in the formation of a partial chromocenter (Fig 3B and 3C). In addition, most of the regions have a clear banding pattern that helps the identification of this chromosome. Prominent landmarks of this chromosome are the characteristic constriction between sections 1 and 2, the puffs in sections 4, 7 and 17 and a series of dark bands in sections 9–11 and 13–15. These regions together with sections 1 and 22 are the most characteristic landmarks that are easily identified in well-spread nuclei (Figs 10 and 11).

### Chromosome III, sections 23–44 (Fig 6)

**Fig 12:**
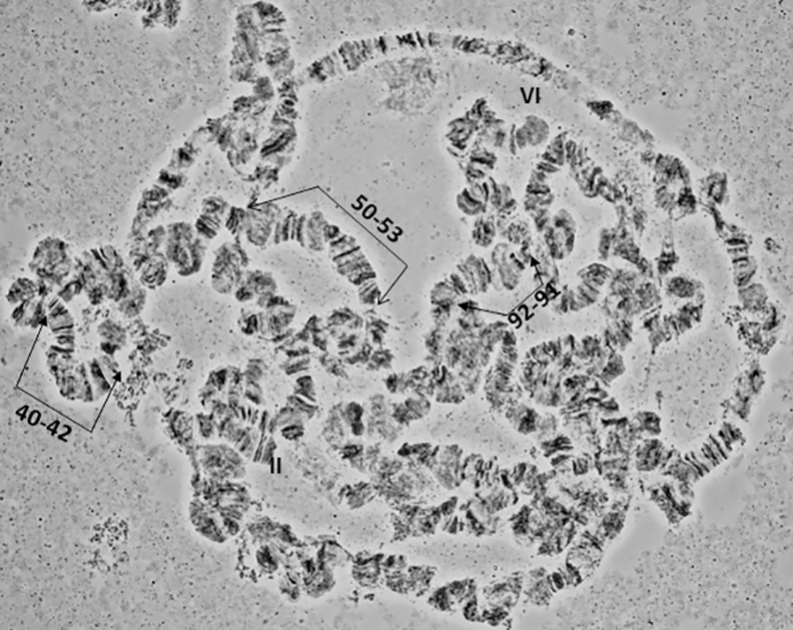
A polytene nucleus of *A*. *fraterculus*. Characteristic landmarks of different polytene chromosome arms are shown. Sections 40–42, 50–53 and 91–92 are indicated. II and VI telomeres are indicated.

Chromosome III presents a poor banding pattern and numerous weak points along most of its length, especially for sections 25–34, making it thus difficult to work with. The telomere in section 23, which is very often involved in ectopic pairing, and section 24, are easily identifiable markers for this chromosome. Sections 35–44 have a better banding pattern and can serve as important landmarks for this chromosome. The end of the region in section 44 usually carries a specific heterochromatic mass that represents the centromeric region of this chromosome (Figs 10 and 12).

### Chromosome IV, sections 45–64 (Fig 7)

Chromosome IV is the most distinctive polytene element of the species with a unique banding pattern starting from the tip in section 45 to section 55 which is easily identified. The most characteristic area is the one included in sections 50–51, with two puffs and a series of bands between them. The telomere is very often taking part in ectopic pairing with other telomeres of the complement (Figs 10 and 12). The centromeric region, section 64, is very difficult to identify and can rarely be observed in spread nuclei. Similarly, difficulties exist in identifying sections 56–64.

### Chromosome V, sections 65–84 (Fig 8)

The telomere of chromosome V, at section 65, has a unique banding pattern and it is very easily identified. Like the other telomeres of the species, it participates in ectopic pairing with other telomeres (Fig 4A). The region close to centromere, section 84, has also a characteristic banding pattern with some diffuse bands and weak points. This end is usually connected with other centromeric regions or participates in a partial chromocenter (Fig 3B). Characteristic landmarks of this chromosome are sections 66–69, section 75 where a characteristic puff is followed by three bands and the two puffs in sections 82 and 83 (Fig 10). In some of the preparations, region 81–84 presented a different banding pattern, probably to differential puffing. Although such variations are often and usually not presented, the fact that the specific one was near the centromere, which is characteristic for the chromosome, made us present this alternative configuration (Fig 8).

### Chromosome VI, sections 85–100 (Fig 9)

Chromosome VI is the smallest chromosome of the complement and the most difficult to work with. It has a poor banding pattern, along with many constrictions and weak points where it is frequently broken. However, there are regions that can be used as diagnostic landmarks for this element. The telomere is localized at the beginning of section 85, based on the characteristic ectopic pairing with other tips observed in several nuclei (Fig 4A and 4C). Additional diagnostic regions of this chromosome are the puffs in sections 86 and 89 and two characteristic ones in sections 91–92 (Figs 11 and 12). It is worth saying that these two last puffs have maintained their structure in all tephritids analyzed so far.

### Comparison of polytene chromosome maps between *A*. *fraterculus* and *A*. *ludens*

Having constructed the polytene chromosome maps of *A*. *fraterculus* we attempted their comparison with the available maps of *A*. *ludens* [68]. Both species have acrocentric chromosomes and the comparison of banding patterns of polytene elements between them revealed several similarities. The telomeres, as well as the centromeres, are either identical or similar between the two species. In both species the telomeres are participating in ectopic pairing between the chromosomes making their identification easy. Moreover, the centromeres are usually connected with heterochromatic threads and very often participate in a partial chromocenter.

**Fig 13:**
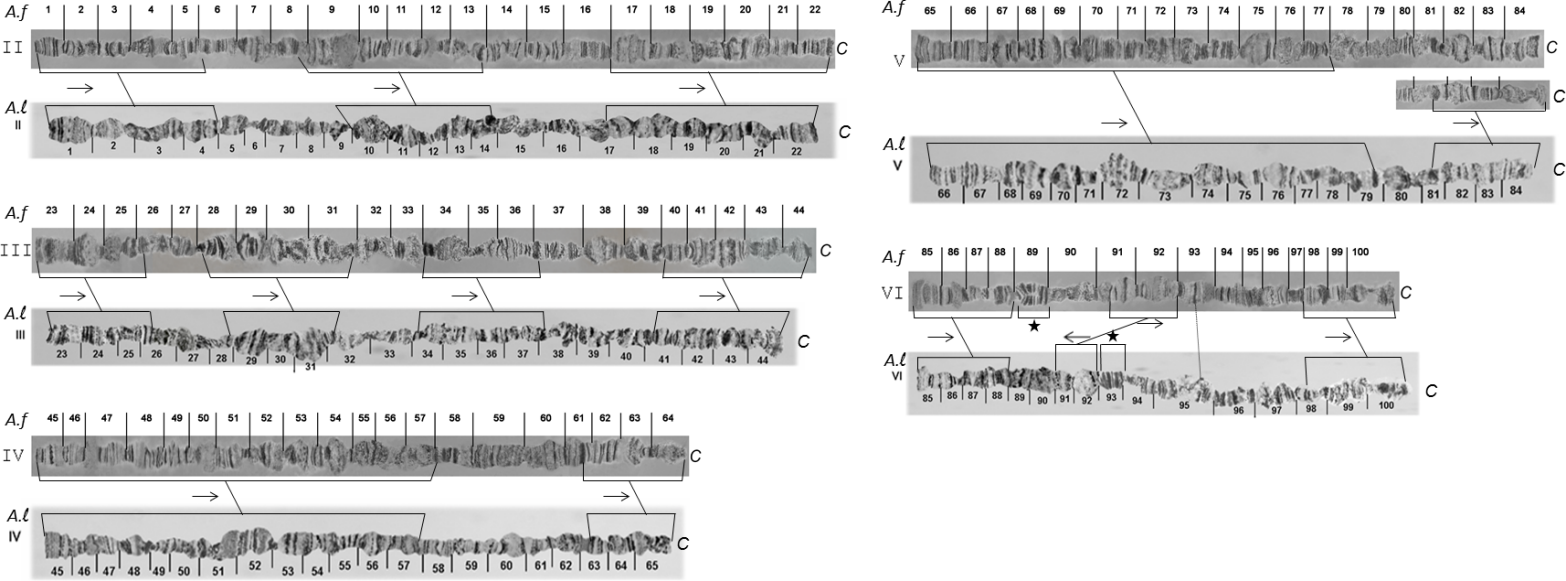
Comparison between *A*. *fraterculus (A*. *f)* and *A*. *ludens (A*. *l)* polytene chromosome maps. Lines connecting the chromosomes indicate sections with similar banding pattern and horizontal arrows show the relative orientation between them. C indicates the centromere. Asterisks indicate the transposition of a specific region between the two species.

The similarity of banding patterns between the two species is remarkable, especially for certain chromosomal regions distributed to all chromosomes, facilitating therefore the establishment of their homologies (Fig 13). Although this comparison is a preliminary one and also difficult due to the poor banding patterns of several chromosomal regions, differences have been observed in the VI polytene chromosome, including a transposition (*A*. *fraterculus Af*. *sp*.*1* section 89, *A*.*ludens* section 93) and an inversion (sections 91–92) (Fig 13). It is interesting that this inversion covers a chromosomal region harboring two characteristic puffs. This chromosomal region (91–92) is found in all tephritids analyzed so far and is polymorphic regarding its position and/or direction within this chromosomal element.

## Discussion

The majority of Tephritidae species analyzed so far exhibit a diploid chromosome number of 2n = 12, including a XX/XY sex chromosome pair. This is the case also for the *A*. *fraterculus* strain analyzed here (Fig 1A–1E). Sex chromosomes are easily identified based on Giemsa staining and C-banding and on the different degree of chromatid separation at metaphases in comparison to the autosomes. These characteristics support the heterochromatic nature of the sex chromosomes, a phenomenon that is common in the different genera of tephritids analyzed so far, namely *Anastrepha*, *Bactrocera*,*Ceratitis*, *Dacus* and *Rhagoletis* [10,65,69–76]. The heterochromatic nature of both sex chromosomes in tephritids is also evident by the abundance of highly repetitive DNA [77–79] and the limited number of genes, including the ribosomal DNA genes mapped on both sex chromosomes. This pattern of localization of the ribosomal genes is common to all tephritids analysed, such as *C*. *capitata* [80], *B*. *oleae* [81], *C*. *rosa* [77], *R*. *pomonella* [82] as well as in *A*. *fraterculus* [45,59]. Additional genes mapped on sex chromosomes include the maleness factor on Y chromosome [78] and *ceratotoxins* that were mapped on the X chromosome of *C*. *capitata* by *in situ* hybridization [83].

The karyotype presented here is in full agreement with previous studies on the Argentinian population of *A*. *fraterculus* characterized as *Af*. *sp*. *1* member of the complex [32,34,45,57,84]. The karyotypes of the seven entities (*Af*. *sp*. *1–7)* identified in *A*. *fraterculus* complex, even though they present the same total number of chromosomes, they can be differentiated mainly by the size and banding pattern of the sex chromosomes [32,45]. Such differences have been reported to differentiate members of other Tephritid species complexes, such as *Bactrocera tau* [85] and *B*. *dorsalis* [86–89]. The size of sex chromosomes among the Tephritid species is variable [63]. This could be the result of the accumulation or loss of heterochromatin in these chromosomes. Such phenomena have been also reported in several *Drosophila* species, including the Hawaiian *Drosophila*, where species exhibit accumulation of heterochromatin on the dot chromosome (microchromosome), thus altering it to rod-shaped [90].

All the members of the *A*. *fraterculus* intrageneric group analyzed so far are characterized by the rod (acrocentric) chromosomes of their mitotic karyotype [32,34,45,57,68]. However, outside this group, there are *Anastrepha* species presenting: i) total chromosome number 2n = 12 with submetacentic or a combination of submetacentric and rod ones and ii) different number of total chromosomes, such as *A*. *pickeli* with 2n = 8 (XX/XY), *A*. *leptozona* with 2n = 10 (XX/XY) or different number of sex chromosomes, such as *A*. *bistrigata* and A. *serpentina* with a karyotype of 2n = 11 for males and 2n = 12 for females (X_1_X_2_Y/X_1_X_2_X_1_X_2_) [66,84]. Such observations are not restricted in the *Anastrepha* genus. Extended studies in several groups of *Drosophila* using comparative mitotic and polytene chromosome analysis revealed that chromosome rearrangements, such as inversions and transpositions as well as fusions/or fissions of chromosome elements have resulted in species-specific chromosomes [2]. Recently, Craddock and colleagues [91] suggested that the frequent changes on the karyotypes within the Hawaiian *Drosophila* species are related with the expansion of their genome size, a phenomenon that most likely has been driven by the addition of heterochromatin and satellite DNA. Such additions resulted in longer acrocentric chromosomes, changing the dot to acrocentric ones, or to metacentric by the addition of a hetrochromatic arm.

In *A*. *fraterculus* polytene nuclei, five long banded polytene chromosomes that represent the five acrocentric autosomes of the metaphase karyotype were found. This is in full agreement with the results from *A*. *ludens* [68], the phylogenetically closest species analyzed so far. In accordance with Tephritidae analyzed so far, sex chromosomes do not form polytene elements, probably due to their under-replication (reviewed in [63]). The sex chromosomes in the polytene nuclei are represented by a granular heterochromatic network (Fig 2). This correlation between sex chromosomes and the granular network in *C*. *capitata* was first suggested by Bedo [92], after analyzing polytene chromosomes of trichogen cells derived from male pupae. Later on [10] this correlation was further established through the analysis of Y–autosome translocations in medfly. More recently, Drosopoulou and her colleagues [81] proved that this network is formed by the sex chromosomes. To do so, they used FISH of sex chromosome specific probes, generated through laser microdissection of the respective mitotic sex chromosomes. Another common feature of tephritids is the absence of a typical chromocenter where all chromosomes are connected through their centromeres. This was also observed in *A*. *fraterculus* where the identification of the centromeric regions presented additional difficulties due to its acrocentric chromosomes. In some cases more than one chromosomes were connected forming a partial chromocenter (Fig 3B and 3C), a situation found also in other tephritids [68,69,76]. Telomeres show ectopic pairing (Fig 4), a phenomenon also observed in several Tephritid species [65,68,69,75,76]. This is probably related to the molecular structure and organization of the distal parts of the chromosomes in these species. In *D*. *melanogaster*, the distal parts of chromosomes consist of specific terminal repeat retrotransposons (Het-A and TART) that are arranged in tracts of variable length among several strains, resulting thus in the extension of the chromosomal ends and the frequent ectopic pairing between telomeres [5,93].

Polytene chromosomes of the two *Anastrepha* species studied so far, *A*. *ludens* and *A*. *fraterculus*, show significant similarities in their banding pattern. In fact, certain chromosomal regions distributed to all elements show the same banding patterns, thus allowing the establishment of chromosomal homologies between the two species (Fig 13). A previous comparative analysis of polytene elements between *C*. *capitata* and *A*. *ludens* showed that chromosome homology between them can also be established [68]. In fact, telomeres and centromeric areas, as well as specific chromosomal regions of each chromosome present the same or very similar banding patterns among the tephritids.

Polytene chromosomes have been used in many taxa to clarify either the status of species complexes or to establish phylogenetic relationships in a significant number of Diptera. The vast majority of such studies refers to *Drosophila* species [2,4,5,94] and mosquitos [3,95–100].

Sturtevant and Novitski [101] revealed the homology of the six chromosomal elements within *Drosophila*, named A-F by Muller [102]. The conservation of the basic elements between *C*. *capitata* and *Drosophila* [13,103] as well as between *B*. *oleae* and *Drosophila* [104] was shown by *in situ* hybridization on polytene chromosomes. Moreover, the chromosome homology between several *Bactrocera* species and *C*. *capitata* as well as *A*. *ludens* and *C*. *capitata* has been established based on both their polytene chromosome banding pattern similarities and/or *in situ* hybridization of selected probes [11,13,68–70,75,76,105–107]. These studies showed that the species are differentiated by fixed chromosomal rearrangements, mainly paracentric inversions, and are characterized by transpositions on specific chromosomes. In addition, two pericentric inversions were found to differentiate *Ceratitis* and *Bactrocera* genera, one of which differentiates *Ceratitis* and *Dacus* [105]. Recently, the genome assembly of *B*. *tryoni* confirmed the above results and showed that the Muller’s elements have maintained their essential identity in both lines of drosophilids and tephritids although a large number of intra-chromosomal rearrangements have occurred. Moreover their data support that X chromosome of Tephritid species is originated from the dot chromosome 4 (Element F) of *Drosophila*. These data clearly support that no new chromosomes and specifically chromosome ends have been created in these insect lineages [108]. Similar conservation of chromosome ends has been observed in mosquito *Anopheles gambiae*, suggesting that this is a common feature of all Diptera [109]. Mason and colleaques [110] showed that Diptera are the only group that lacks telomerase and this is a factor that contributes to their chromosome ends stability. These species protect their chromosome ends by the recruitment of retrotransposons [108].

Chromosomal rearrangements, mainly inversions, are believed to be a key player in speciation of Diptera [4,94]. The role of chromosome inversions in speciation is being discussed for decades and recent models suggest that they can promote speciation through the suppression of recombination within the inversion and near the inversion breakpoints that subsequently leads to the restriction in gene flow [111–115]. The presence of at least one fixed paracentric inversion in chromosome VI that differentiates *A*. *fraterculus* from *A*. *ludens* (Fig 13), two *Anastrepha* species belonging to the same intrageneric group, is in line with the aforementioned model of Diptera speciation.

Centromeres and telomeres have long been characterized as dynamic regions of chromosomal evolution. Several studies in primates indicate that the centromere position can change during short periods of evolutionary time. There are different models that try to explain the repositioning of centromeres. This can be done either through transposition of centromeric regions to new chromosomal regions or by the *de novo* emergence of centromeres in new regions (neocentromere emergence) [116–119]. According to the first model, this repositioning is the result of chromosomal rearrangements that could explain this change (sequential pericentric inversions, for example). All Tephritid species analyzed so far, present metacentric or submetacentric autosomes, with the exception of the *Anastrephas* that present acrocentric ones. Assuming that the first model of centromere repositioning applies, chromosomal changes such as transpositions or pericentric inversions should be evident and explain the transformation from acrocentric to metacentric chromosomes or *vice versa*. However, the comparison of the polytene chromosome banding pattern of the two *Anastrepha* species with acrocentric autosomes (*A*. *fraterculus* and *A*. *ludens*) to all other tephritids (with metacentric and submetacentric autosomes) does not support the presence of such extended rearrangements. The similarity in the banding pattern of chromosomal ends (meaning telomeres of the metacentric chromosomes and telomeres–centromeres of the acrocentric chromosomes) support the stability of the chromosome ends. Therefore, the *de novo* formation of neocentromeres in specific chromosomal regions is more compatible with our data. As discussed before, *Anastrephas* are variable both in chromosome number and metaphase configuration of chromosomes. The availability of so diverse chromosome configurations shows that polytene chromosome analysis of *Anastrepha* species with different metaphase karyotypes could shed light to the centromere evolution in Tephritidae and further elucidate their phylogenetic relationships.

## Conclusions

The first polytene chromosome maps for *Anastrepha fraterculus* (*A*.*sp1*) presented here and their future comparison to the polytene chromosomes of other members of the complex may reveal additional structural differences among them as well as their phylogenetic relationships. The comparison with the polytene chromosome maps of *A*. *ludens* shows that these maps can be used in comparative studies with other *Anastrepha* species as well. Polytene chromosome analysis constitutes an important component for the development and characterization of stable GSSs of *A*. *fraterculus* towards the supporting of SIT control methods in the species. Finally, any future research on the construction of genome assemblies for *A*. *fraterculus* could benefit by *in situ* hybridization of unique genes or sequences on polytene chromosomes.
